# Case report: A Boy with an inability to walk; do not forget about scurvy

**DOI:** 10.51866/cr.590

**Published:** 2024-05-14

**Authors:** Emira Mansor Noor Emilia, Abdul Hadi Said

**Affiliations:** 1 MBBCh, Department of Family Medicine, International Islamic University Malaysia, Jalan Sultan Ahmad Shah, Bandar InderaMahkota, Kuantan, Pahang, Malaysia. Email: emiliaemira@iium.edu.my; 2 MD, MMed Family Medicine, Department of Family Medicine, International Islamic University Malaysia, Jalan Sultan Ahmad Shah, Bandar InderaMahkota, Kuantan, Pahang, Malaysia.

**Keywords:** Scurvy, Lower limb pain, Nutritional deficiency, Autism spectrum disorder

## Abstract

Scurvy, a condition caused by vitamin C deficiency, is characterized by a syndrome of multisystem disorder due to defective collagen production and antioxidative function. This condition is infrequent in this modern era; thus, it is often not within the list of differential diagnoses. The broad clinical picture is generally overlooked as other systemic illnesses, resulting in an extensive investigation that delays the diagnosis. Herein, we report a case of an 8-year-old boy with underlying autism spectrum disorder who presented with lower limb pain and other constitutional symptoms. Examination revealed multiple hyperpigmented scars over the upper and lower limbs and gingival hyperpigmentation. With history of picky eating habits and clinical symptoms supported by radiographic findings, scurvy was suspected and subsequently confirmed based on a low level of ascorbic acid. With vitamin C supplementation and proper nutritional support, the patient recovered well.

## Introduction

Since the human body cannot synthesise vitamin C, dietary supplements are the only source of this vitamin. Around 1500 BC, Egyptians first documented scurvy, a condition caused by vitamin C deficiency. Owing to nutrition and food supplementation developments, scurvy has been largely eradicated in wealthy nations.^1^ The clinical manifestations of scurvy might mimic those of viral infections, haematological malignancies, and systemic disorders.^2^ Thus, the diagnosis of scurvy is frequently delayed or missed altogether, leading to extensive laboratory and radiographic investigations and, possibly, severe complications. Children with autism spectrum disorder (ASD) are more susceptible to scurvy and other micronutrient deficiencies due to their selective diets and smaller food selections.^3^ This case report aimed to create awareness about the risk of scurvy in children with a restricted diet to avoid unnecessary invasive investigations and procedures and prevent further serious complications.

## Case presentation

FH, an 8-year-old boy with underlying ASD, presented to the emergency department with lower limb pain for 1 week. The pain began in both ankles and ascended to the bilateral lower limbs. It was persistent and worsened with movement and manipulation. Initially, the pain was mild and partially relieved by rest. However, as the pain worsened, the patient lost the ability to walk, and the pain began to interfere with his everyday tasks. He could not score the pain but reported that the pain was worse in the morning. There was no history of trauma or falls before the symptom onset. There was no easy bruising, delayed wound healing, or gum bleeding. Upon further history-taking, the patient was also noted to have intermittent fever for the past 3 months. He had poor appetite and significant weight loss, amounting to 3 kg within 3 months. He also had multiple absences from school due to fever and tiredness. The patient had a few clinic visits for intermittent fever, but the diagnosis was uncertain, and he was treated only symptomatically.

The patient was diagnosed with ASD at 3 years of age but had not received proper follow-up. Apart from mild ASD, he had no known other medical illnesses. He lived with his parents and three other siblings. He was the second child. All his siblings were healthy. His father was a labourer, and his mother was a housewife who provided home-cooked meals for him. However, his parents noted that he had been a picky eater since 3 years of age. He disliked fruits and vegetables. He was selective in his food choices, eating only a plate of rice with fried chicken or fish. His mother provided meals to his liking because he threw tantrums when the menu differed.

His antenatal and postnatal histories were unremarkable. His immunisations were up to age. Regarding family history, his paternal aunts were diagnosed with leukaemia and systemic lupus erythematosus.

**Figure 1:**
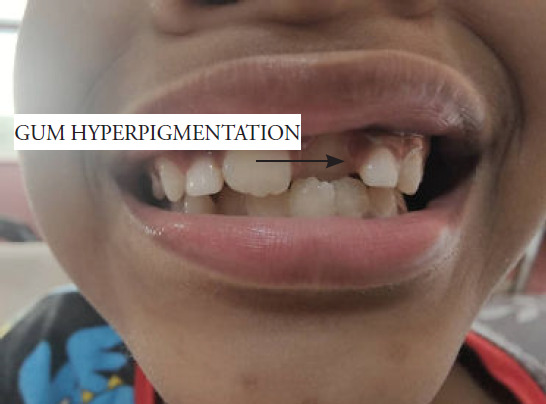
Gum hyperpigmentation.

**Figure 2:**
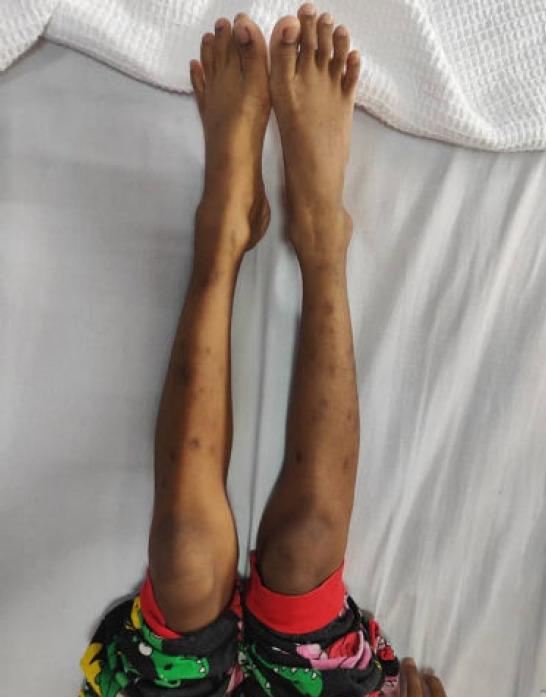
Hyperpigmented scars on the lower limbs.

On examination, the patient was alert but appeared cachexic and had mild pallor. He was afebrile. His heart rate was 101 beats per minute; blood pressure, 99/54 mmHg; respiratory rate, 22 breaths per minute; and SpO2 level, 99% on room air. He had no brittle or spoon-shaped nails. His weight and height were slightly lower than the 3rd centile, as charted in the growth chart. His oral hygiene was poor, with gum hyperpigmentation noted (Figure 1). No oral ulcer, gum hypertrophy, or bleeding was noted. Multiple hyperpigmented scars were found over the bilateral upper and lower limbs (Figure 2). The inguinal lymph nodes were palpable. Examination of the lower limbs showed an antalgic gait. The strength of both lower limbs was grade 4/5, with normal tone, reflex, and sensation. No noticeable joint swelling or skeletal deformity was noted. The range of movement of the bilateral lower limbs was normal. There was no spinal deformity, and other systems had unremarkable findings.

**Figure 3:**
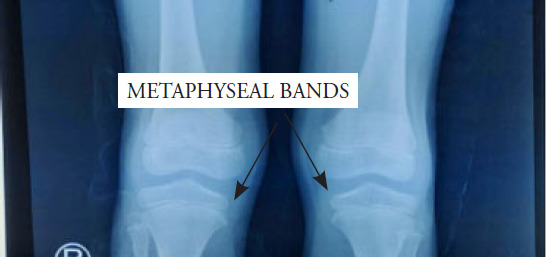
Lower limb radiograph showing metaphyseal bands.

Based on the presenting history and physical findings, the initial working diagnosis was either a rheumatological disorder, such as juvenile idiopathic arthritis, or haematological malignancy, such as leukaemia. Hence, the initial work-up was geared towards these two possibilities.

The full blood picture showed hypochromic microcytic anaemia, suggesting possible iron deficiency anaemia. Screening for malignancy showed no blast cells. The tumour lysis result was also normal. Chest radiography revealed no mediastinal mass or any infective changes. Antinuclear antibody and rheumatoid factor were negative in the evaluation for connective tissue disease. The erythrocyte sedimentation rate Erythrocyte Sedimentation Rate (ESR) and C-reactive protein level were high at 117 mm/h and 44.7 mg/L, respectively. Radiography of both lower limbs showed ill-defined metaphyseal bands at the distal metaphysis of the bilateral femur (Figure 3). There was no metaphyseal widening. The bones had normal density. There was neither fracture nor periosteal reaction.

Scurvy was suspected based on the clinical manifestations: bone pain, gum hyperpigmentation, multiple hyperpigmented scars over the lower limbs, selective diet history, and metaphyseal bands detected on lower limb radiography. This was confirmed based on a low level of vitamin C (<5 mmol/L). Upon diagnosis, he was started on oral supplementation with vitamin C 100 mg thrice daily. He also received multivitamins and underwent physiotherapy to reduce his lower limb pain.

His joint pain improved after vitamin C supplementation, and his fever was resolved on day 2 of admission. He could ambulate and was not limping before he was discharged. He was discharged after 6 days of admission, with oral vitamin C 300mg daily for 2 weeks. He was referred for nutritional rehabilitation and physiotherapy for regular exercise and muscle strengthening. Marked improvement was noted during follow-up at the clinic 2 weeks after discharge. His appetite had improved, and he had gained 2 kg. The joint pain considerably lessened. He was not on vitamin C maintenance because all symptoms had resolved. Regarding diet modification, he occasionally drank fruit juices but ate plenty of blended vegetables mixed with rice or noodles.

## Discussion

Ascorbic acid, or vitamin C, is essential in multiple biochemical pathways and is obtained through diet. It plays various roles, including enhancing iron absorption, acting as an antithrombotic or antioxidant, improving immune function, initiating carnitine production, helping wound healing, and stimulating steroid and catecholamine syntheses.^4^

The clinical spectrum of scurvy varies. Patients usually present with cutaneous manifestations such as easy bruising, delayed wound healing, and gum bleeding, as well as anaemic symptoms. Paediatric patients commonly experience musculoskeletal problems such as joint pain, limb swelling, limping, joint swelling, or myalgia. In patients with prolonged vitamin C deficiency, osteoporosis is also possible.^5^ In the present case, the patient exhibited lower limb pain, gum hyperpigmentation, lower limb hyperpigmentation, and intermittent fever. Initially, these symptoms were suspected to indicate a rheumatological pathology or haematological malignancy.

In developed and developing countries, scurvy is rare due to abundant food supplies rich in vitamin C. However, in such contexts, suspicion of scurvy should arise in patients with developmental or behavioural issues such as ASD, food malabsorption and swallowing disorders. These patients may face challenges obtaining adequate amounts of vitamin C from their diet.^6^ In a study conducted in Malaysia, many children with ASD had atypical eating behaviours, and more than half of these children had oral sensory processing problems, thus increasing their risk of nutritional deficiency.^7^

Radiography indicated that scurvy was present in the metaphysis of all long bones and was more prominent in the lower limbs. A clear metaphyseal band named the ‘Trummerfeld zone,’ a marked white line corresponding to the thickened zone of calcification called the ‘white line of Frankel,’ and an irregular metaphyseal margin known as ‘Pelkan spur’ were noted.^8^

The diagnosis of scurvy can be confirmed with a serum ascorbic acid level of <0.2 mg/dL (10 μmol/L). It is often delayed or overlooked because of the condition's rarity and can cause unnecessary workups.^9^ The treatment of scurvy is simple and safe with daily supplementation of 300–1000 mg of vitamin C for 1–3 months, and symptoms usually resolve within days to weeks of treatment. Complete recovery is anticipated after approximately 3 months of regular vitamin C supplementation. This potentially fatal disease can be prevented with as low as 10 mg of ascorbic acid per day, an amount easily obtained through fresh fruits and vegetables. The recommended daily requirement for children is 30 mg/day; adolescents 65 mg/day; and adults 70 mg/day.^10^

Our case also highlights the importance of managing picky eating habits among children, particularly those with ASD. This requires commitment from the whole family and support from dieticians for nutritional assessment and dietary counselling. Parents and caretakers should ensure their children have a balanced diet to prevent vitamin deficiency. Occupational therapy and physiotherapy play a significant role in improving patients’ functional independence, reducing pain, and enhancing mobility. Follow-up appointments with paediatricians or specialists in ASD management are necessary to address patients’ overall health, well-being and developmental needs.

## Conclusion

The suspicion rate for scurvy remains low in Malaysia; thus, scurvy is commonly overlooked or misdiagnosed. Thorough history-taking, proper clinical examination, and correct radiographical interpretation are necessary to diagnose scurvy in children. Healthcare providers should have a higher suspicion of vitamin deficiency in patients with selective eating habits.

## References

[1] Gandhi M, Elfeky O, Ertugrul H, Chela HK, Daglilar E (2023). Scurvy: Rediscovering a Forgotten Disease.. Diseases..

[2] Alqanatish JT, Alqahtani F, Alsewairi WM, Al-kenaizan S (2015). Childhood scurvy: an unusual cause of refusal to walk in a child.. Pediatr Rheumatol Online J..

[3] Liuzzo Scorpo M, Corsello G, Maggio MC (2021). Scurvy as an Alarm Bell of Autistic Spectrum Disorder in the First World: A Case Report of a 3-Year-Old Girl.. Am J Case Rep..

[4] Dosedel M, Jirkovsky E, Macakova K (2021). Vitamin C-Sources, Physiological Role, Kinetics, Deficiency, Use, Toxicity, and Determination.. Nutrients..

[5] Agarwal A, Shaharyar A, Kumar A, Bhat MS, Mishra M (2015). Scurvy in pediatric age group - A disease often forgotten?. J Clin Orthop Trauma..

[6] Nastro A, Rosenwasser N, Daniels SP (2019). Scurvy Due to Selective Diet in a Seemingly Healthy 4-Year-Old Boy.. Pediatrics..

[7] Zulkifli MN, Kadar M, Hamzaid NH (2022). Weight Status and Associated Risk Factors of Mealtime Behaviours among Children with Autism Spectrum Disorder.. Children (Basel)..

[8] Chalouhi C, Nicolas N, Vegas N (2020). Scurvy: A New Old Cause of Skeletal Pain in Young Children.. Front Pediatr..

[9] Khalife R, Grieco A, Khamisa K, Tinmouh A, McCudden C, Saidenberg E (2019). Scurvy, an old story in a new time: The hematologist's experience.. Blood Cells MolDis..

[10] Ministry of Health Malaysia. (2017). RECOMMENDED NUTRIENT INTAKES for MALAYSIA RNIA Report of the Technical Working Group on Nutritional Guidelines National Coordinating Committee on Food and Nutrition Ministry of Health Malaysia Ministry of Health Malaysia..

